# Activation of Myd88-Dependent TLRs Mediates Local and Systemic Inflammation in a Mouse Model of Primary Sjögren's Syndrome

**DOI:** 10.3389/fimmu.2019.02963

**Published:** 2020-01-09

**Authors:** Jeremy Kiripolsky, Rose-Anne Romano, Eileen M. Kasperek, Guan Yu, Jill M. Kramer

**Affiliations:** ^1^Department of Oral Biology, School of Dental Medicine, State University of New York at Buffalo, Buffalo, NY, United States; ^2^Department of Biostatistics, School of Public Health and Health Professions, State University of New York at Buffalo, Buffalo, NY, United States; ^3^Department of Oral Diagnostic Sciences, School of Dental Medicine, State University of New York at Buffalo, Buffalo, NY, United States

**Keywords:** NOD.B10, decorin, B cell, salivary gland, TLR2, TLR4

## Abstract

Toll-like receptors (TLRs) are important mediators of chronic inflammation in numerous autoimmune diseases, although the role of these receptors in primary Sjögren's syndrome (pSS) remains incompletely understood. Previous studies in our laboratory established Myd88 as a crucial mediator of pSS, although the disease-relevant ligands and the upstream signaling events that culminate in Myd88 activation have yet to be established. The objective of this study was to identify specific Myd88-dependent TLR-related pathways that are dysregulated both locally and systemically in a mouse model of pSS [NOD.B10Sn-*H2*^*b*^/J (NOD.B10)]. We performed RNA-sequencing on spleens derived from NOD.B10 mice. We then harvested salivary tissue and spleens from *Myd88*-sufficient and deficient C57BL/10 (BL/10) and NOD.B10 mice and performed flow cytometry to determine expression of Myd88-dependent TLRs. We cultured splenocytes with TLR2 and TLR4 agonists and measured production of inflammatory mediators by ELISA. Next, we evaluated spontaneous and TLR4-mediated inflammatory cytokine secretion in NOD.B10 salivary tissue. Finally, we assessed spontaneous Myd88-dependent cytokine secretion by NOD.B10 salivary cells. We identified dysregulation of numerous TLR-related networks in pSS splenocytes, particularly those employed by TLR2 and TLR4. We found upregulation of TLRs in both the splenic and salivary tissue from pSS mice. In NOD.B10 splenic tissue, robust expression of B cell TLR1 and TLR2 required Myd88. Splenocytes from NOD.B10 mice were hyper-responsive to TLR2 ligation and the endogenous molecule decorin modulated inflammation via TLR4. Finally, we observed spontaneous secretion of numerous inflammatory cytokines and this was enhanced following TLR4 ligation in female NOD.B10 salivary tissue as compared to males. The spontaneous production of salivary IL-6, MCP-1 and TNFα required Myd88 in pSS salivary tissue. Thus, our data demonstrate that Myd88-dependent TLR pathways contribute to the inflammatory landscape in pSS, and inhibition of such will likely have therapeutic utility.

## Introduction

Primary Sjögren's syndrome (pSS) is an autoimmune disease characterized by exocrine gland dysfunction and immune hyperactivity. Patients with pSS typically experience loss of saliva and tear production as well as serious systemic sequelae including pulmonary and renal pathoses and B cell lymphoma (1). Salivary gland inflammation is considered a hallmark of the disease (2), although the inciting disease events and pathways that maintain chronic inflammation in this tissue remain unknown. Several studies suggest that activation of innate immunity precedes the adaptive response in pSS (3–8). However, the specific pathways that lead to disease initiation and progression are poorly understood.

Recent work in our laboratory identified a key role for the adaptor Myeloid differentiation primary response protein 88 (Myd88) in pSS pathogenesis (9). Myd88 is critical for both innate and adaptive immune function, as most Toll-like receptor (TLR) and Interleukin-1 Receptor (IL-1R) family members require Myd88 for signal transduction (10). Using a pSS mouse model that lacked *Myd88* (NOD.B10^*Myd*88−/−^), we found spontaneous activation of Myd88-dependent pathways is crucial for disease progression (9). While TLR and IL-1 family members are increased in murine and human SS salivary tissue (3, 11–16), relatively few studies have examined the functional contribution of these pathways to salivary and systemic inflammation and most of these have focused on the Myd88-independent agonist TLR3 (5, 6, 17–21).

To understand the contribution of Myd88-dependent TLR pathways to pSS pathogenesis, we first performed RNA-sequencing (RNA-seq) on splenic tissue from NOD.B10Sn-*H2*^*b*^/J (NOD.B10) females with clinical disease and C57BL/10 (BL/10) controls. We found numerous genes related to TLR activation were altered, including those utilized in TLR2 and TLR4-mediated signaling cascades. We found TLR2, TLR2 co-receptors (TLR1 and TLR6), and TLR4 levels were altered in splenic and salivary B cells derived from pSS mice. These findings carried functional significance as splenocytes from NOD.B10 females with clinical disease were hyper-responsive to TLR2 ligation. Moreover, treatment of pSS splenocytes with the damage-associated molecular pattern (DAMP) Decorin (Dcn) resulted in altered inflammatory cytokine secretion in a TLR4-dependent manner. Of note, Dcn induced a distinct inflammatory profile as compared to lipopolysaccharide (LPS) in NOD.B10 splenocytes. Furthermore, we found TLR4 ligation of pSS salivary cells resulted in the production of inflammatory mediators, and this was particularly pronounced in tissues derived from female animals as compared to males. Finally, pro-inflammatory cytokine secretion was attenuated in salivary gland tissue from *Myd88*-deficient NOD.B10 mice. These data demonstrate that specific Myd88-dependent TLRs are dysregulated in pSS and identify Dcn as a novel mediator of inflammation in disease. Blockade of TLR pathways may represent an innovative therapeutic strategy for the treatment of local and systemic disease manifestations.

## Materials and Methods

### Mice

Age-matched male and female NOD.B10 and age-matched female BL/10 mice were used (Jackson Laboratory, Bar Harbor ME, USA). NOD.B10^*Myd*88+/−^ and NOD.B10^*Myd*88−/−^ mice were previously described (9). B6.129P2(SJL)-*Myd88*^*tm*1.1*Defr*^/J mice were purchased from Jackson Laboratories. We generated BL/10 mice that are deficient in *Myd88* by crossing BL/10 mice with *Myd88*^−/−^ mice (B6.129P2(SJL)-*Myd88*^*tm*1.1*Defr*^/J). We then bred 10 successive generations of heterozygotes back to BL/10 mice to generate *Myd88*-deficient mice that are fully BL/10 (BL/10^*Myd*88−/−^). Animals were bred and maintained at the University at Buffalo and housed in identical conditions. All animals used in this study were at least 26 weeks of age, the time that NOD.B10 females normally develop clinical disease (22, 23). Mice were cared for and handled in accordance with the institutional animal care and use committee (IACUC) and US NIH guidelines.

### RNA Sequencing

Spleens were isolated from NOD.B10 females at 26 weeks of age and from age and sex-matched BL/10 controls. RNA was isolated according to standard protocols using a Qiagen RNeasy kit. RNA meeting quality standards (>8.0) were used as input for RNA-seq using Illumina'sTru-seq low-throughput multiplex protocol. Three biological replicates for each strain were sequenced in parallel using an Illumina HiSeq 2500, with 50 cycle single-end read reactions. Sequence reads were mapped to the reference genome sequence of *Mus musculus* (mm10 build) using Tophat2 (PMID 23618408). Reads that aligned to the mouse genome were counted using featureCounts. We compared the BL/10 and NOD.B10 strains to identify the Differentially Expressed Genes (DEGs) using the DESeq pipeline in the DESeq2 R package (24). The DESeq2 package provides methods to test for differential expression using the negative binomial generalized linear models. The estimates of dispersion and logarithmic fold changes incorporate data-driven prior distributions. In this analysis, we used BL/10 mice as the reference group and identified the set of DEGs with the Benjamin–Hochberg adjusted *p* < 0.05. Datasets have been deposited in the Gene Expression Omnibus (GEO) database under the accession number GSE136402.

### Isolation and Culture of Salivary and Splenic Tissue

Submandibular gland (SMG) tissue and spleens were harvested following euthanasia. For RNA isolation, tissue was snap frozen in liquid nitrogen. For primary splenocyte culture experiments, spleens were mechanically disrupted and red cell lysis was carried out using ACK lysing buffer (Gibco). Cells (5 × 10^6^ per well) were plated in 0.5 mL of complete RPMI media containing 2% FBS and cultured for 24 h in media alone, or with media containing *S. aureus* peptidoglycan (PGN) (1.25 μg/ml) (Invivogen), Pam3SCSK4 (P3C4) (5 ng/ml) (Invivogen), FSL-1 (5 ng/ml) (Invivogen), *S. typhimurium*, (0.25 μg/ml) (Sigma-Aldrich), *E. coli* LPS B5-Ultrapure (B5-UP) (0.1 μg/ml) (Invivogen), or murine Dcn (20 μg/mL, R&D systems). TAK-242 was used to inhibit TLR4 activation (5 μM, EMD Millipore). Finally, polymyxin B (PMB) was used at a concentration of 100 μg/mL (Invivogen). Supernatants were harvested and stored at −20°C until use.

For primary SMG culture experiments, tissue was dispersed enzymatically and mechanically in dispersion buffer [(DMEM-Ham's F12 (1:1), bovine serum albumin (1%), CaCl_2_ (0.2 mM) (ThermoFisher Scientific), hyaluronidase (400 U/mL) (Sigma-Aldrich), and collagenase P (0.08 mg/mL) (Worthington Biochemical, Lakewood, NJ, USA)] for 30 min and incubated in a shaking water bath at 37°C. Cells were washed twice in acini buffer (pH 7.4) (NaCl (120 mM), KCl (4 mM), KH_2_PO_4_ (1.2 mM), MgSO_4_ (1.2 mM), HEPES (15 MM), dextrose (10 mM), CaCl_2_ (1 mM), and bovine serum albumin (1%) (ThermoFisher Scientific), and plated in complete media as previously described (25). Where indicated, cells were cultured in the presence or absence of LPS derived from *S. minnesota* (10 μg/mL) for 24 h (Sigma-Aldrich). Supernatants were harvested and stored at −20°C until use.

### Multiplex Cytokine Array

Supernatants from BL/10, NOD.B10, NOD.B10^*Myd*88+/−^, and NOD.B10^*Myd*88−/−^ SMG tissue and splenocytes were harvested as described above. Bradford assays were used to confirm total protein concentrations were similar in all samples (BioRad). Multiplex cytokine arrays were performed (Quansys Biosciences). Each sample was analyzed in triplicate.

### IL-6 and TNFα ELISAs

Splenocyte supernatants were harvested following culture for 24 h and IL-6 and TNFα levels were quantified by ELISA (Invitrogen and BioLegend, respectively). All samples were analyzed in duplicate in accordance with manufacturer instructions.

### Flow Cytometry

Flow cytometry was performed as previously described (9). Briefly, SMG cells and splenocytes were dissociated as described above. Cells were incubated in Fc block [(Cd16/32, clone 2.4G2), BD Biosciences] and were treated with the following antibodies as indicated: B220 (clone RA3-6B2, BD Biosciences), TLR1 (clone CB225, BD Biosciences), TLR2 (clone 6C2, BD Biosciences), TLR4/Md2 (Clone MTS510, BD Biosciences), and TLR6 (clone C1N2, BD Biosciences). Data were acquired using a Fortessa (BD Biosciences) and analyzed using FlowJo software (BD Biosciences).

### Statistics

All statistical analyses were performed using GraphPad Prism software. Data were analyzed by Mann–Whitney or one-way ANOVA with the Tukey's multiple comparisons tests as indicated.

### BarGraph

Gene ontology bargraph was generated in R (26), using the ggplot2 (27) package. The top 1,000 differentially enriched genes in NOD.B10 spleens were used as input for gene ontology analysis on the DAVID website version 6.8 (28, 29) using default settings. The KEGG (30–32) pathway output was then imported into R and re-formatted to generate the resulting bargraph.

## Results

### TLRs and TLR-Related Pathways Are Dysregulated in NOD.B10 Mice

**Figure 1:**
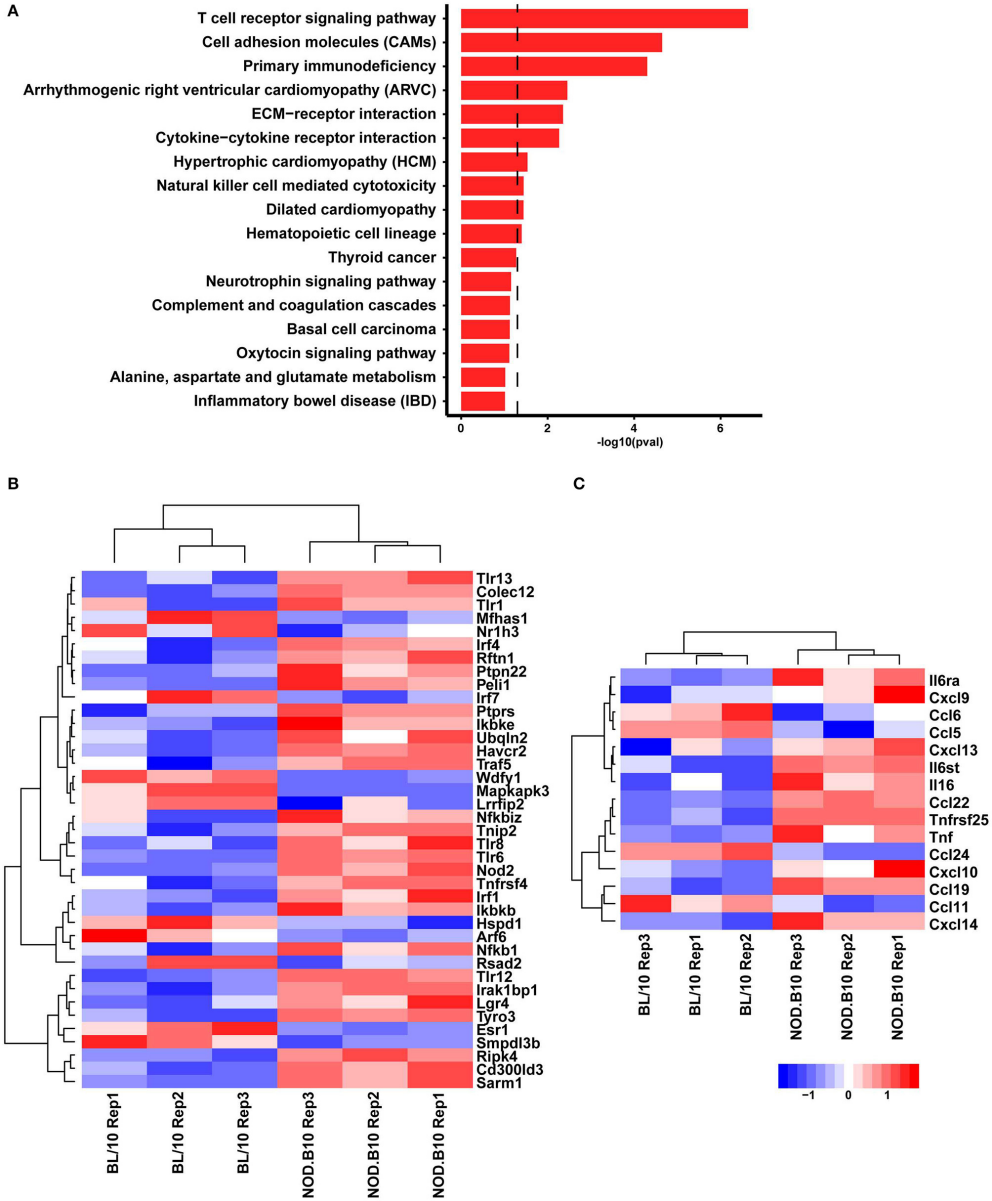
TLR-related genes are dysregulated in splenic tissue derived from NOD.B10 mice. Spleens were harvested from NOD.B10 females with clinical disease (*n* = 3) and age and sex-matched BL/10 controls (*n* = 3) and RNA-sequencing was performed. Bargraph showing the KEGG pathway terms represented in the top 1000 genes enriched in NOD.B10 mice. Dotted line represent the boundary for *p* = 0.05 **(A)**. Heatmap visualizations showing the relative expression levels of selected DEGs involved in TLR-related signaling pathways **(B)** and cytokines and chemokines **(C)** that were enriched in NOD.B10 splenic tissue.

In order to better define the genetic differences between pSS mice and healthy controls, we performed RNA-seq based expression profiling of spleens isolated from female NOD.B10 mice with clinical disease and age and sex-matched BL/10 controls (*n* = 3 each). Differential gene expression analysis focusing on the top 1,000 genes enriched in the NOD.B10 mice compared to control animals revealed enrichment of genes associated with immune activation, including T cell receptor signaling pathways, cytokine-cytokine receptor interactions and ECM (extra-cellular matrix)-receptor interactions (Figure 1A). We next mined our RNA-seq dataset and identified a number of DEGs associated with TLR-related signaling pathways (Figure 1B) and cytokines and chemokines that are expressed as a consequence of TLR activation (Figure 1C). These data indicate a role for dysregulated TLR signaling pathways in the pathogenesis of pSS.

### Expression of TLR1, TLR2, TLR6, and TLR4 Is Altered in Splenic and Salivary Tissue From NOD.B10 Females

**Figure 2:**
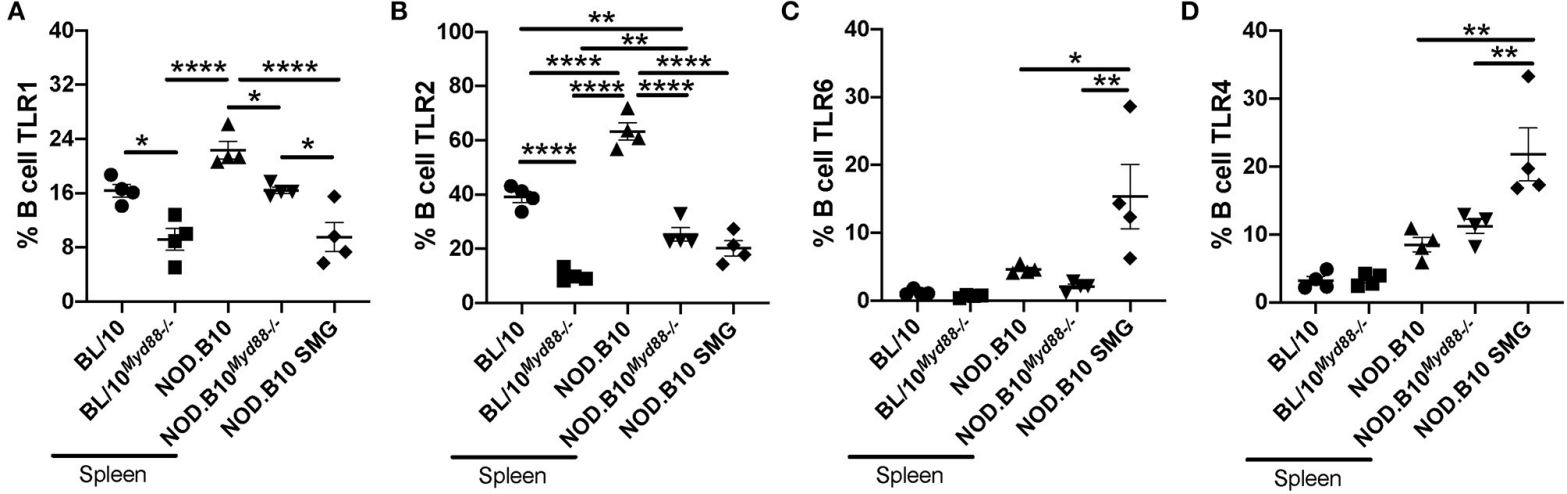
TLR expression is increased in splenic B cells from NOD.B10 mice in a Myd88-dependent manner and differs from that seen in salivary B cells. Spleens were isolated from female BL/10, BL/10^*Myd*88−/−^, NOD.B10, and NOD.B10^*Myd*88−/−^ mice (*n* = 4 each) and SMG tissue was isolated from NOD.B10 females (*n* = 4 each). Flow cytometry was performed to assess expression of **(A)** TLR1, **(B)** TLR2, **(C)** TLR6, and **(D)** TLR4 on B220+ B cells. Significance was determined using a one-way ANOVA with the Tukey's multiple comparisons tests. Mean and SEM are shown (^*^p ≤ 0.05, ^**^p ≤ 0.01, ^****^p ≤ 0.0001).

Since we found that genes associated with TLR2 and TLR4 signaling were altered in NOD.B10 splenic tissue, we performed flow cytometry to evaluate expression of these receptors. We examined TLR2 and its co-receptors TLR1 and TLR6, as well as TLR4 on splenic B cells derived from BL/10, BL/10^*Myd*88−/−^, NOD.B10^*Myd*88−/−^, and NOD.B10 females (*n* = 4 each). We focused our analyses on B cells because B cell-intrinsic TLR expression is critical to the development and progression of other autoimmune diseases (33–35). We found that levels of TLR2 were significantly increased on NOD.B10 splenic B cells as compared to those derived from BL/10 controls (*p* < 0.0001) (Figure 2B). We then assessed levels of these receptors in the *Myd88*-deficient strains. We found that the percentage of splenic B cells expressing TLR1 and TLR2 was diminished significantly in BL/10^*Myd*88−/−^ animals compared with the parental strain (*p* = 0.02 and *p* < 0.0001, respectively) (Figures 2A,B). Furthermore, the percentage of splenic B cells expressing TLR2 was diminished significantly in NOD.B10^*Myd*88−/−^ animals compared to NOD.B10 mice (*p* < 0.0001) (Figure 2B). Significantly, the percentage of NOD.B10^*Myd*88−/−^ B cells expressing TLR1, TLR6, and TLR4 were similar to the BL/10 control strain (Figures 2A,C,D) and the percentage of B cells expressing TLR2 was diminished in NOD.B10^*Myd*88−/−^ females as compared to the healthy BL/10 animals (*p* = 0.009) (Figure 2B). Thus, the percentage of splenic B cells expressing TLR2 and TLR2 co-receptors is higher in NOD.B10 animals as compared to BL/10 controls. Moreover, *Myd88*-deficient NOD.B10 splenic B cells demonstrate TLR1, TLR2, and TLR6 expression levels that are similar to or even lower than those observed in B cells derived from healthy BL/10 controls. We then examined TLR4/MD2 expression and observed no difference in B cell TLR4 expression between the *Myd88*-deficient animals from either strain as compared to the parental controls.

Finally, we examined TLR expression on NOD.B10 B cells in SMG tissue. Of note, we could not compare these levels to B cells derived from salivary tissue of BL/10 controls, as these animals do not have significant B cell infiltration of salivary tissue at 26 weeks of age (22). Interestingly, we found salivary B cells from NOD.B10 mice expressed lower levels of TLR1 and TLR2 than splenic B cells (*p* < 0.0001 for each comparison) (Figures 2A,B), while the percentage of B cells expressing TLR6 and TLR4 was elevated in B cells derived from salivary B cells as compared to those from splenic tissue (*p* = 0.2 and *p* = 0.001, respectively) (Figures 2C,D). These findings suggest TLR expression is dysregulated in B cells in pSS and may be influenced by tissue-specific cues within the microenvironment.

### Splenocytes Derived From NOD.B10 Mice Are Hyper-Responsive to TLR2 Ligation

**Figure 3:**
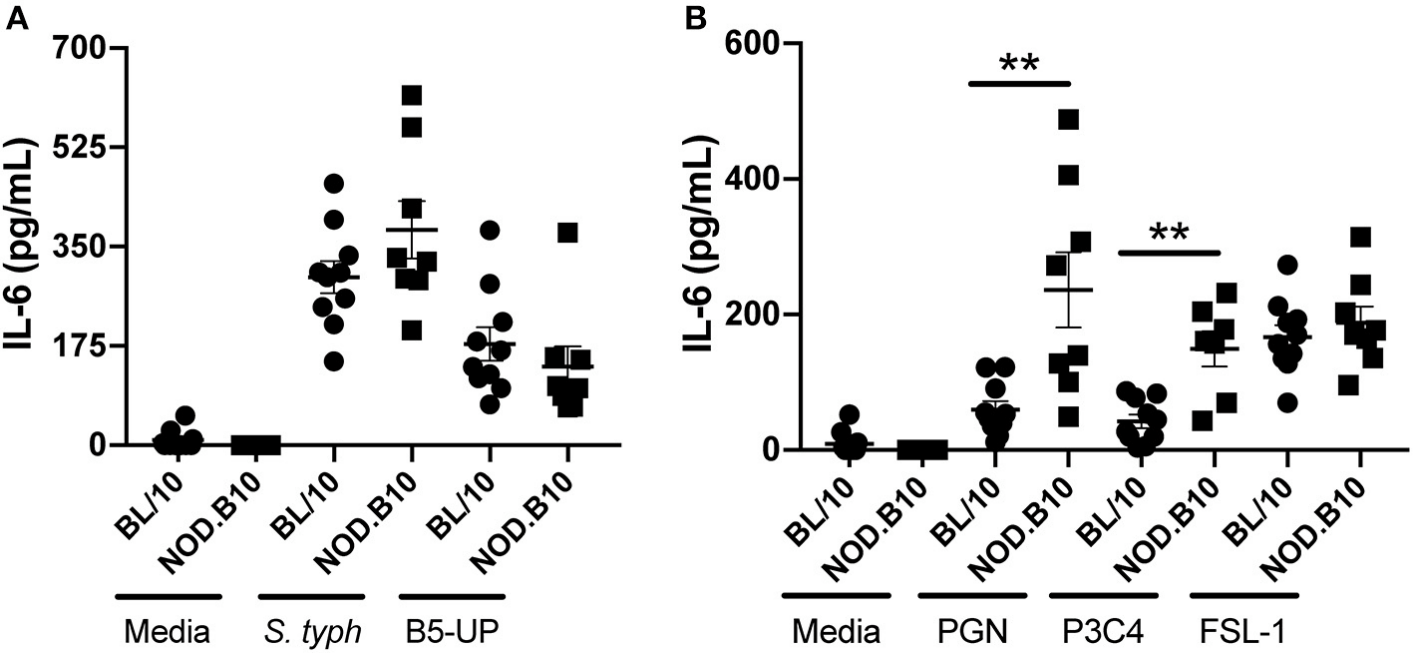
NOD.B10 splenocytes are hyper-responsive to TLR2 ligation. Spleens were isolated from female NOD.B10 mice with clinical disease (*n* = 8) and age and sex-matched BL/10 controls (*n* = 10). Splenocytes were cultured as indicated, supernatants were collected, and IL-6 ELISAs performed. All samples were analyzed in duplicate and results of two independent experiments are shown. Significance was determined using the Mann-Whitney test. Mean and SEM are shown (^**^p ≤ 0.01).

We next sought to determine whether the heightened expression of TLR2, TLR2 co-receptors, and TLR4 in splenic B cells carried functional significance. To this end, we first harvested splenocytes from NOD.B10 females with clinical disease and age and sex-matched BL/10 controls. We incubated cells with 2 different TLR4 agonists. We used LPS derived from *S. typhimurium*, an agonist that primarily activates TLR4 (36). We also stimulated cells with an ultrapure LPS that only activates TLR4 (B5-UP derived *E. coli* 055:B5) (37). We found that both agonists resulted in robust IL-6 secretion from BL/10 and NOD.B10 splenocytes, although there were no differences observed between the 2 strains when cells were stimulated with either *S.typhimurium* or B5-UP LPS (*p* = 0.3 and 0.2, respectively) (Figure 3A).

We then stimulated splenocytes with TLR2 agonists. We first used PGN derived from *S. aureus*. This ligand primarily activates TLR2, although it is also reported to signal via NOD1 and NOD2 (38). Therefore, to examine activation of specific TLR2 binding partners, we stimulated cells with Pam3CSK4, a synthetic triacylated lipopeptide that activates TLR1/TLR2 heterodimers or FSL-1, a synthetic diacylated lipopeptide that has specificity for the TLR6/TLR2 heterodimer (39, 40). We found that both PGN and Pam3CSK4 stimulation resulted in heightened IL-6 secretion in splenocytes derived from NOD.B10 animals as compared to BL/10 controls (*p* = 0.002 and 0.009, respectively) (Figure 3B). Interestingly, we saw no difference in IL-6 production when we stimulated cells with FSL-1 (*p* = 0.6), suggesting that this TLR2 hypersensitivity was primarily a result of TLR1/TLR2 ligation rather than TLR2/TLR6 interactions (Figure 3B). Therefore, NOD.B10 splenocytes are hyper-responsive to TLR2 agonists.

### The DAMP Dcn Modulates Inflammatory Cytokine Production in a TLR4-Dependent Manner

**Figure 4:**
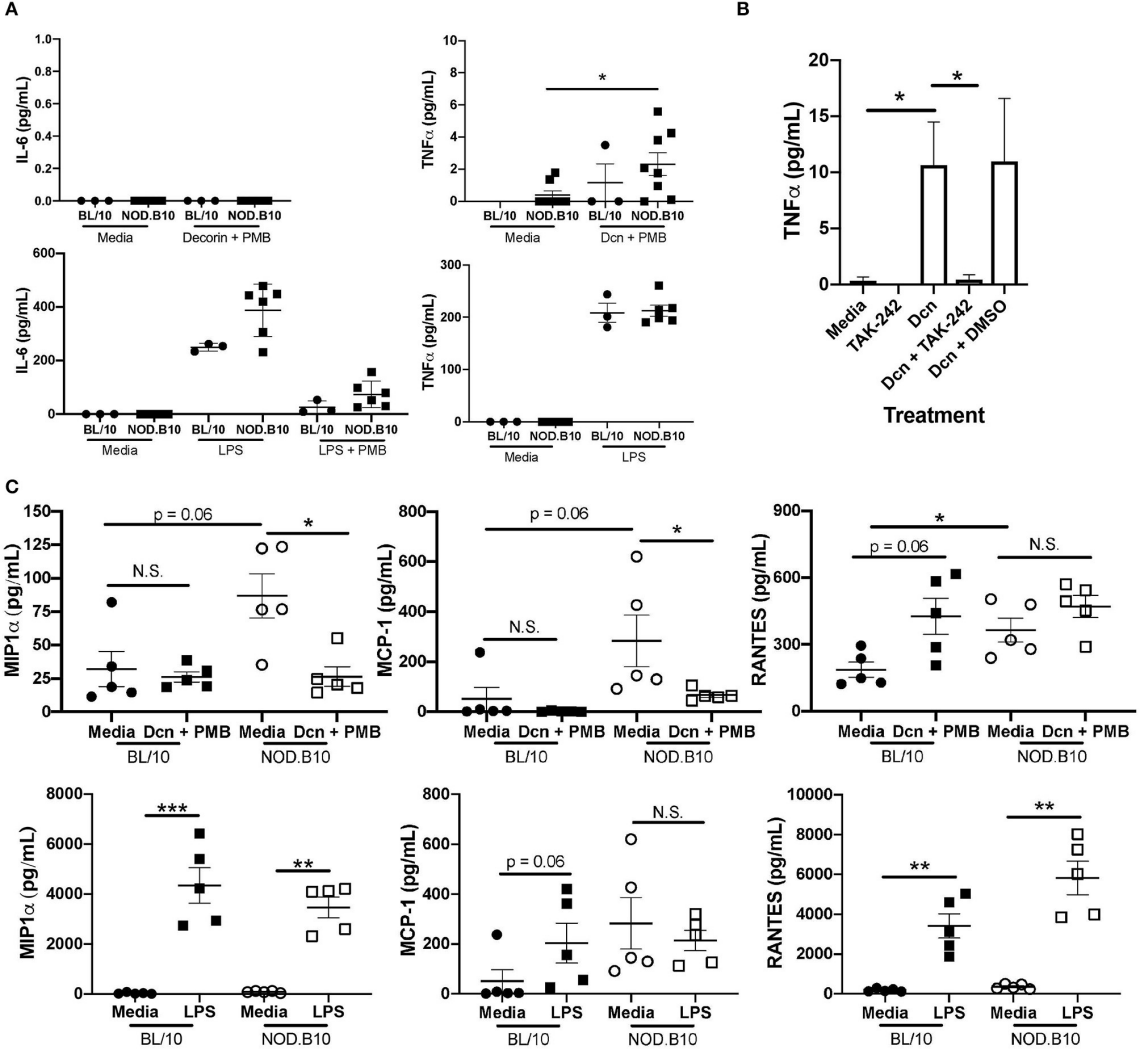
Dcn and LPS induce distinct inflammatory outcomes in NOD.B10 splenocytes via TLR4. Splenocytes were isolated from NOD.B10 females (*n* = 6) with clinical disease and age and sex-matched BL/10 controls (*n* = 3). **(A)** Cells were cultured in media alone, Dcn and PMB, or *S. typhimurium* LPS alone or with LPS and PMB. Supernatants were harvested and assayed for IL-6 and TNFα by ELISA. **(B)** Splenocytes from NOD.B10 females with clinical disease were cultured with the indicated inhibitors and controls and the supernatants harvested. TNFα was measured by ELISA. Combined results of at least 3 independent experiments are shown. All samples were analyzed in duplicate. **(C)** Splenocytes were isolated from NOD.B10 females (*n* = 5) with clinical disease and age and sex-matched BL/10 controls (*n* = 5). Cells were cultured in media alone, Dcn and PMB, or *S. typhimurium* LPS for 24 h. Supernatants were harvested and MIP-1α, MCP-1, and RANTES levels were quantified by cytokine multiplex array. All samples were evaluated in triplicate. Significance was determined using the Mann–Whitney test. Mean and SEM are shown (^*^p ≤ 0.05, ^**^p ≤ 0.01, ^***^p ≤ 0.001, N.S., non-significant).

Although pSS patients exhibit increased incidence of infections compared to the general population, it is likely that TLR activation in pSS is not solely mediated by microbial infection (41). Therefore, we sought to determine whether DAMPs promote inflammatory cytokine secretion and activate lymphocytes in the context of pSS. To this end, we stimulated splenocytes with the DAMP Dcn, as this ECM molecule is degraded in salivary tissue from NOD.B10 mice with disease (42). To confirm that this effect was not a result of endotoxin contamination, cells were treated concomitantly with PMB. We found that incubation of BL/10 and NOD.B10 splenocytes with Dcn resulted in secretion of TNFα, but IL-6 production was not seen (Figure 4A). We also analyzed LPS (*S. typhimurium*) treated cells and observed robust IL-6 and TNFα secretion as expected (Figure 4A). We then sought to determine whether Dcn-mediated TNFα production was dependent on TLR4. We incubated cells with the TLR4 inhibitor TAK-242 (43) and found that treatment with TAK-242 abrogated TNFα secretion (Figure 4B). Of note, incubation with a TLR2 neutralizing antibody in the presence of Dcn did not diminish TNFα secretion (data not shown).

To determine whether Dcn induced secretion of additional pro-inflammatory cytokines in the context of pSS, we harvested splenocytes from NOD.B10 females with clinical disease and those derived from age and sex-matched BL/10 mice (*n* = 5 each). Cells were incubated with media alone, Dcn in combination with PMB, or *S. typhimurium* LPS and cytokine multiplex arrays were performed on the supernatants (Figure 4C). Strikingly, we found that Dcn reduced production of both Macrophage Inflammatory Protein-1α (MIP-1α, also called CCL3) and Monocyte Chemotactic Protein-1 (MCP-1, also called CCL2) in splenocytes derived from NOD.B10 females (*p* = 0.02 and 0.02, respectively). In contrast, MIP-1α secretion by NOD.B10 splenocytes was increased in the presence of LPS (*p* = 0.008), while secretion of MCP-1 by NOD.B10 splenic cells did not differ between cells treated with media or LPS (*p* = 0.8). Of note, these findings were specific for pSS, as there was no difference in production of MIP-1α or MCP-1 between BL/10 cells treated with media alone or Dcn (*p* = 0.7 and 0.1, respectively). Finally, we examined RANTES (also termed CCL5) secretion in BL/10 and NOD.B10 splenocytes. We found that Dcn promoted RANTES secretion in BL/10 splenocytes as compared to cells incubated with media alone, although the difference did not reach significance (*p* = 0.06). Interestingly, Dcn did not alter RANTES secretion in NOD.B10 splenocytes (*p* = 0.2), although RANTES levels were elevated in supernatants derived from unstimulated NOD.B10 splenic cells as compared to those derived from BL/10 cells animals (*p* = 0.03). Of note, RANTES was induced in both BL/10 and NOD.B10 splenocytes treated with LPS (*p* = 0.008 and 0.008, respectively). Finally, Dcn did not alter secretion of IL-1α, IL-1β, IL-2, IL-4, IL-5, IL-10, IL-12p70, or IL-17 in either BL/10 or NOD.B10 splenocytes (Supplemental Figure 1). Thus, Dcn-mediated signals are distinct from those induced by LPS in NOD.B10 splenocytes, and TLR4 activation by decorin may be an important endogenous mediator of inflammation in pSS.

### Strong Constitutive and LPS-Induced Cytokine Secretion Is Observed in SMG Tissue Isolated From Female NOD.B10 Mice

Next, we sought to determine whether NOD.B10 salivary cells exhibited spontaneous and TLR4-induced cytokine secretion. We harvested SMG tissue from female NOD.B10 mice with clinical disease (*n* = 3). SMG tissue from age-matched NOD.B10 males was used as a control (*n* = 3), since slower disease progression is observed in males (23). Cells were pooled and cultured for 24 h in the presence or absence of LPS and the supernatant harvested.

**Figure 5:**
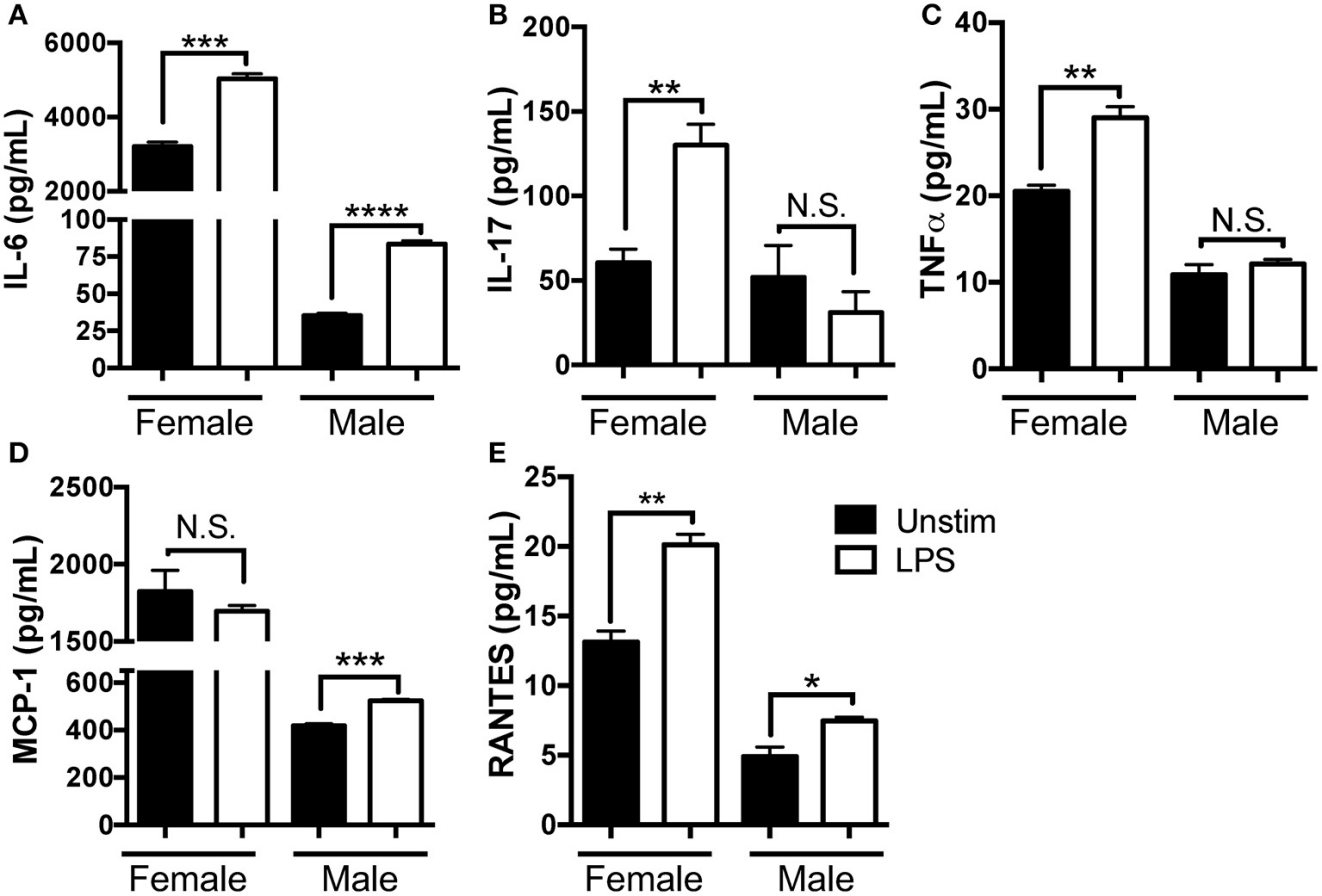
NOD.B10 SMG tissue exhibits spontaneous secretion of pro-inflammatory mediators that are enhanced in the presence of LPS. NOD.B10 female mice with clinical disease (*n* = 5) were euthanized and SMG tissue harvested and pooled. Cells were cultured in the presence or absence of LPS for 24 h and the supernatant harvested. **(A)** IL-6, **(B)** IL-17, **(C)** TNFα, **(D)** MCP-1, and **(E)** RANTES were assessed by cytokine multiplex array. All samples were analyzed in triplicate and results from one of three independent experiments are shown. Significance was determined using Mann–Whitney test. SEM is shown (^*^p ≤ 0.05, ^**^p ≤ 0.01, ^***^p ≤ 0.001, ^****^p ≤ 0.0001, N.S., non-significant).

We found female SMG cells secreted higher spontaneous levels of IL-6, IL-17, MCP-1, TNFα, and RANTES as compared to those derived from males and we observed little spontaneous inflammatory cytokine expression in the male SMG cells. LPS treatment resulted in heightened IL-6, IL-17, TNFα, and RANTES in female salivary tissue. When stimulated with LPS, male SMG cells were also responsive, secreting elevated levels of IL-6, MCP-1, and RANTES, although levels secreted were reduced as compared to females. Importantly, female SMG cells were hyper-responsive to LPS stimulation, as levels of IL-17 and TNFα increased in SMG tissue derived from females, but did not change in the male samples (Figure 5). Statistical analyses from 3 independent experiments are shown in Table 1. Taken together, these data show that NOD.B10 SMG cells secrete pro-inflammatory cytokines spontaneously and this secretion is further enhanced in the presence of LPS. Moreover, the effect of TLR4 agonism is more pronounced on female SMG tissue as compared to male.

**Table 1:** Statistical analyses of NOD.B10 multiplex array results (Figure 5).

**Cytokine**	**Female**	**Male**	**Unstim**	**LPS**
	**Unstim vs. LPS**	**Unstim vs. LPS**	**Female vs. Male**	**Female vs. Male**
IL-6	• *p* = 0.005 • *p* = 0.3 • *p* < 0.0001	<0.0001 <0.0001 N.D.	<0.0001 <0.0001 <0.0001	<0.0001 <0.0001 <0.0001
IL-17	• *p* = 0.009 • *p* = 0.001 • *p* = 0.1	0.4 0.4 N.D.	0.7 0.01 0.03	0.005 <0.0001 0.005
TNFα	• *p* = 0.04 • *p* = 0.004 • *p* < 0.0001	0.4 0.02 N.D.	0.002 <0.0001 <0.0001	0.0002 <0.0001 <0.0001
MCP-1	• *p* = 0.4 • *p* = 0.03 • *p* = 0.01	0.0006 0.009 N.D.	0.0005 <0.0001 <0.0001	<0.0001 <0.0001 <0.0001
RANTES	• *p* = 0.003 • *p* = 0.0003 • *p* = 0.002	0.03 0.03 0.2	0.002 <0.0001 0.001	<0.0001 <0.0001 0.03

*1, 2, and 3, independent experiments; N.D., none detected*.

### Myd88-Deficient NOD.B10 Mice Exhibit Attenuated Proinflammatory Cytokine Secretion

**Figure 6:**
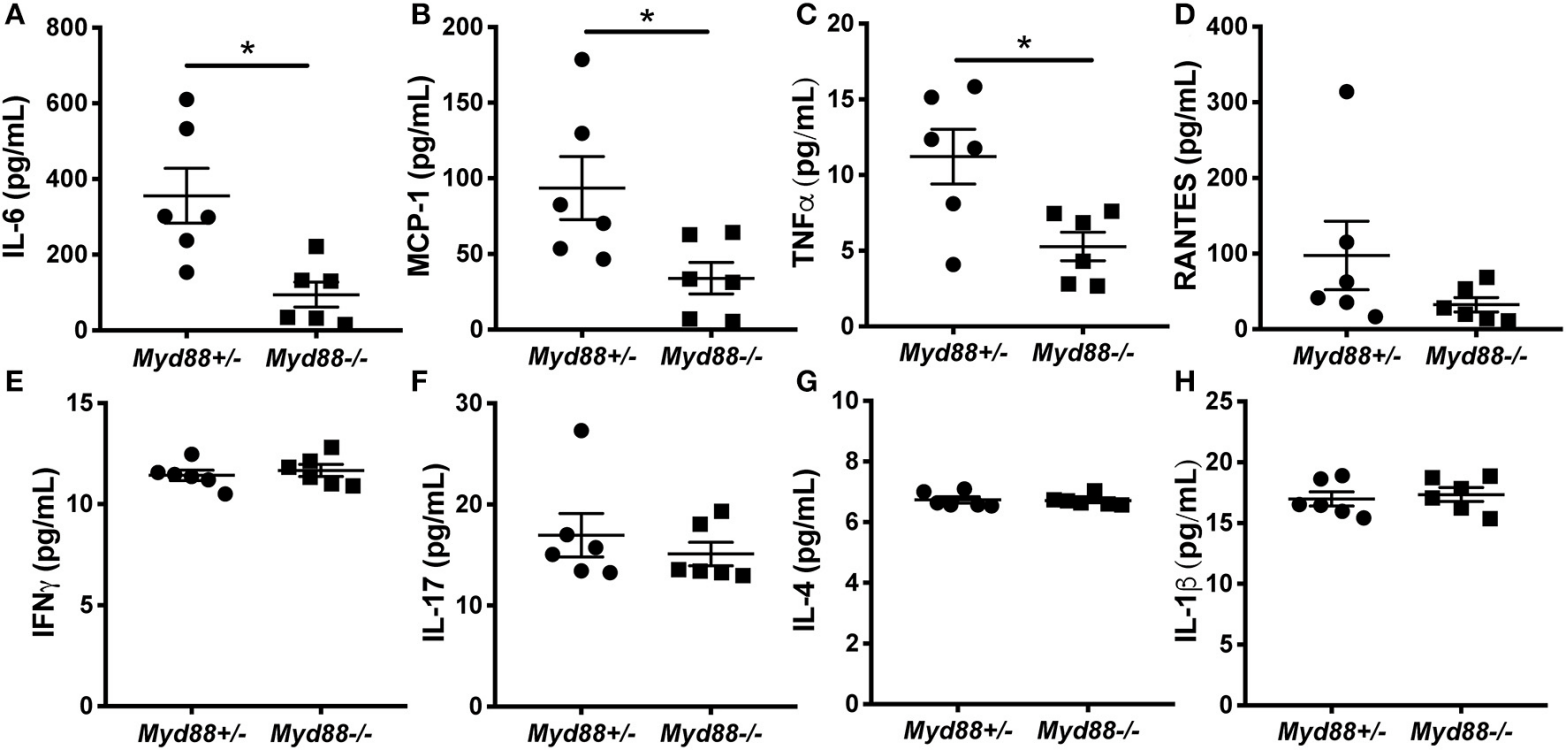
IL-6, MCP-1, and TNFα is decreased in SMG tissue derived from NOD.B10^*Myd*88−/−^ females. SMG tissue was harvested from NOD.B10^*Myd*88+/−^ and NOD.B10^*Myd*88−/−^ mice (*n* = 6 each). Tissue was dissociated, cultured for 24 h, and the supernatant harvested. Cytokine multiplex arrays were performed for **(A)** IL-6, **(B)** MCP-1, **(C)** TNFα, **(D)** RANTES, **(E)** IFNγ, **(F)** IL-17, **(G)** IL-4 and **(H)** IL-1β. Each sample was analyzed in triplicate. Significance was determined using the Mann–Whitney test. Mean and SEM are shown (^*^p ≤ 0.05).

Next, we sought to determine whether the heightened cytokine secretion observed in female SMG tissue was dependent on Myd88. Previous studies in our lab found that salivary gland inflammation is diminished in NOD.B10 females that lack *Myd88* and these animals are protected from loss of salivary flow (9). To determine whether Myd88 was a key mediator of spontaneous inflammatory cytokine production in salivary tissue, we cultured SMG cells from NOD.B10^*Myd*88+/−^ and NOD.B10^*Myd*88−/−^ females and harvested the supernatant. Since NOD.B10 and NOD.B10^*Myd*88+/−^ females secrete similar levels of IL-6 (data not shown) we compared NOD.B10^*Myd*88+/−^ SMG supernatants (*n* = 6) to those of littermate control NOD.B10^*Myd*88−/−^ females (*n* = 6) for the multiplex array studies. We assayed a panel of inflammatory mediators and found that IL-6, MCP-1, and TNFα were decreased in supernatant derived from *Myd88*-deficient mice (*p* = 0.004, 0.03, and 0.03, respectively) (Figures 6A–C). Interestingly, we found no differences in levels of RANTES, IFNγ, IL-17, IL-4 or IL-1β between the NOD.B10^*Myd*88+/−^ and NOD.B10^*Myd*88−/−^ females (Figures 6D–H). These data indicate that Myd88-dependent signaling pathways contribute to the production of specific inflammatory cytokines in the context of pSS.

## Discussion

Previous work by our group demonstrated that Myd88 is essential for pSS disease development in NOD.B10 mice (9). However, the specific signals that rely on Myd88 for induction of inflammation in the context of pSS remained unknown. To identify putative Myd88-dependent pathways that are dysregulated in pSS, we performed RNA-seq and identified dysregulation of many pathways associated with immune activation, including TLRs and TLR-related networks in NOD.B10 spleens. We then examined expression of TLR2, TLR2 co-receptors, and TLR4 and found differences in expression of these receptors in salivary and splenic tissue. Moreover, splenocytes derived from pSS mice exhibited hyper-responsiveness to TLR2 agonists as compared to those from BL/10 controls. Treatment of NOD.B10 splenocytes with the DAMP Dcn modulated inflammatory cytokine production via TLR4. Of note, inflammatory outcomes induced by Dcn were distinct from those mediated by LPS in pSS. We found pSS salivary tissue exhibited spontaneous cytokine secretion that was further enhanced by TLR4 ligation. Finally, production of many of these pro-inflammatory cytokines in salivary tissue required Myd88. Taken together, these data suggest TLR2 and TLR4-mediated signaling networks drive chronic inflammation in the context of pSS.

While the ligands that drive disease progression in pSS remain unknown, activation of TLR signaling pathways by DAMPs plays a definitive role in many autoimmune diseases, including lupus, rheumatoid arthritis, and scleroderma (44–46). DAMPs are endogenous molecules that are released upon necrosis or due to damage from tissue injury (45, 47, 48). In healthy individuals, activation of TLR pathways via DAMPs results in inflammatory gene expression which mediates tissue repair (49). However, in individuals with autoimmunity, activation of these pathways by DAMPs can result in release of pro-inflammatory cytokines which further perpetuate an inflammatory response (45, 47, 49).

Of relevance to the current study, elevated levels of proteolytic degradation of two DAMPs that are normal constituents of the ECM (Dcn and biglycan) are reported in salivary gland tissue from NOD.B10 mice (42). Proteoenzymatic digestion of these DAMPs may release them from the ECM in soluble form, thereby making them accessible as ligands for TLRs (50). This TLR activation may then promote inflammatory cytokine secretion by numerous cell types, ultimately leading to further ECM degradation (49, 51–53). Both TLR2 and TLR4 are activated by numerous DAMPs (48). While a previous study found Dcn induced cytokine secretion via TLR2 and TLR4 in macrophages (54), our work revealed that Dcn signals through TLR4, but not TLR2, to induce TNFα in NOD.B10 splenic tissue. Thus, it is possible that differences exist in signaling cascades activated by Dcn in a cell-type specific manner and these could be altered in the context of pSS.

It is important to point out that the animals examined in this study are 26 weeks of age, the time at which the animals display clinical disease. Several studies demonstrate key differences in the immune response that vary depending on disease stage. Accordingly, data suggest the innate response is activated early in disease, and that activation of the innate response results in engagement of the adaptive (3, 4, 55–58). Thus, it is likely that the role of the Dcn-induced TLR-mediated signaling may vary depending on the disease stage. For example, an inciting disease event may lead to tissue damage within exocrine glands that results in the release of soluble ECM molecules including Dcn. These DAMPs could then bind to TLRs expressed by salivary gland epithelial cells and tissue resident immune populations, thereby driving production of pro-inflammatory mediators and facilitating the recruitment of innate and adaptive cells into the salivary tissue (59). This paradigm is supported by studies of kidney pathoses, as DAMP-mediated recruitment of Th1 and Th17 cells is mediated by TLR2 and TLR4 interactions (60). Since most cells recruited to the salivary tissue express TLRs (8), this may lead to a continuous cycle of unremitting inflammation and further tissue destruction. As tissue breakdown continues, Dcn levels may become elevated systemically, as is observed for the related DAMP biglycan in SLE patients (44), and this may be a mechanism contributing to the systemic disease manifestations observed in pSS. Thus, blockade of these signals early in the course of disease may mitigate both local and systemic disease severity.

Furthermore, our data suggest that tissue-specific TLR expression mediates distinct pSS disease manifestations. We focused our initial analyses on salivary and splenic B cells, as the importance of TLR activation of B cells in other autoimmune diseases is evident (61). In pSS, the significance of B cell-intrinsic TLR signaling is poorly understood (62). Interestingly, expression of TLR4 and TLR6 was higher while levels of TLR1 and TLR2 were diminished in B cells derived from NOD.B10 SMG tissue as compared to B cells derived from the spleen. While the reasons for this are unclear at present, it is possible that differences in the microenvironment of the SMG and the spleen may govern B cell TLR expression in these tissues. Importantly, although the regulation of TLR2 and TLR4 is complex, it is well-established that receptor-ligand interactions initiate downstream signaling pathways that regulate TLR expression in an autocrine manner (51, 53, 63). These TLRs may be up- or downregulated, depending on the specific ligand encountered (51). This elaborate regulatory network provides an elegant feedback loop that allows for the modulation of inflammation. However, in autoimmunity these networks may be triggered inappropriately, thereby leading to chronic activation and subsequent tissue damage.

In pSS, splenic and salivary cells are hyper-responsive to TLR2 and TLR4, respectively, suggesting an important role for DAMP-induced inflammation in disease in both immune cells and salivary epithelium. Our findings are consistent with those in humans, as TLR2, TLR4, and TLR6 are upregulated in salivary tissue of pSS patients and pSS peripheral blood cells are hyper-reactive to ligation by TLR4, TLR2, and TLR2/6 agonists (LPS, peptidoglycan, and zymosan, respectively) (16). Furthermore, our work demonstrates that Dcn modulates inflammation in NOD.B10 splenocytes. These findings have important implications for disease pathogenesis, as it is possible that circulating B cells that have high TLR4 expression could encounter soluble decorin within pSS salivary tissue (16). TLR ligation by this DAMP could then promote inflammation and further tissue destruction through secretion of cytokines such as TNFα and possibly RANTES (16, 53). Interestingly, work in a lupus model demonstrates that overexpression of biglycan, a DAMP that is related to Dcn, induces similar levels of TNFα in spelenic tissues as were observed in our pSS mice (44). It is important to point out that Dcn also attenuated production of specific pro-inflammatory mediators in NOD.B10 splenocytes, as production of both MIP-1α and MCP-1 was mitigated in the presence of Dcn. Thus, further work is needed to understand this potentially dichotomous role of Dcn in pSS.

MIP-1α, MCP-1, and RANTES are important mediators of immune cell chemotaxis. Indeed, MIP-1α is secreted by most hematopoietic cells and binds CCR1 and CCR5. MIP-1α recruits immune cells to sites of inflammation, specifically T cells, monocytes, dendritic cells (DCs), and natural killer (NK) cells (64). More recent work revealed that MIP-1α induces chemotaxis of T follicular regulatory (Tfr) cells and promotes interactions between antigen-specific B cells and Tfr cells (65). MCP-1 is a ligand for CCR2 and is expressed by diverse cell types, including epithelium and macrophages (66). CCL2 drives recruitment of monocytes/macrophages, memory T cells, and NK cells (66). In addition, RANTES is produced by many cells types, including T cells and binds to CCR1, CCR3, and CCR5 to mediate chemotaxis of T cells, DCs, eosinophils, NK cells, and mast cells to inflamed tissues (67). Lastly, a mouse model that overexpresses TNFα in salivary tissue specifically develops robust sialadenitis reminiscent of that seen in SS patients (68). Thus, Dcn-induced dysregulation of cytokines and chemokines in the context of pSS likely alters the immune response, including the recruitment of both innate and adaptive cells to sites of inflammation in disease.

Previous work by our group demonstrated Myd88 is crucial for pSS development in NOD.B10 mice (9). The present study extends these findings, as we demonstrated that Myd88 controls expression of TLR1 and TLR2 in splenic B cells. Furthermore, Myd88 is required, at least in part, for spontaneous production of IL-6, MCP-1, and TNFα within the salivary tissue. Importantly, numerous studies show Myd88 is essential for DAMP-mediated inflammation (44, 60, 69, 70). Our findings suggest that the attenuated disease phenotype observed in the *Myd88*^−/−^ NOD.B10 model may be due to reduced activation of DAMP-mediated TLR signaling networks (9). While further data are needed to identify the specific DAMPs, and whether these are interactions are driven by exocrine-gland destruction specifically, our work suggests that ligation of TLR4 by Dcn may be a novel underlying source of inflammation in pSS.

Here we show LPS and Dcn induce secretion of distinct pro-inflammatory cytokines, as Dcn only induces TNFα, while LPS induces both IL-6 and TNFα. Moreover, LPS induces significant MIP-1α secretion while secretion of this cytokine is diminished by Dcn in NOD.B10 splenocytes (Figure 4). These data are consistent with the literature, as DAMPs and PAMPs that bind the same TLR can induce divergent signaling outcomes (53). Specifically, while ligation of TLR4 with either a DAMP or a PAMP resulted in NF-κB activation in macrophages, ligand-dependent differences were observed in both cytokine secretion and phosphoproteomic profile (53). While the reasons for these differential signaling outcomes are incompletely understood, it is likely that different TLR agonists facilitate recruitment of specific adaptor molecules that direct distinct signaling outcomes. For example, LPS activation of TLR4 requires both MD-2 and CD14, while the DAMP tenascin-C does not (71). Another factor that likely contributes to the specificity of the response is the ligand-induced conformational changes of the TLR itself, as different LPS chemotypes result in the formation of either monomeric or dimeric TLR4 complexes (72). While the aforementioned study did not assess downstream signaling events in depth (72), it is possible that DAMPs and PAMPs may induce distinct conformational changes in cognate TLRs that fine-tune the inflammatory response. Further studies are needed to determine the exact mechanisms governing signaling outcomes by exogenous and endogenous agonists that bind the same TLR.

In conclusion, we established that TLR1, TLR2, TLR6, and TLR4 are differentially expressed in splenic and salivary B cells of NOD.B10 mice and expression of TLR1 and TLR2 is attenuated in the absence of Myd88. Importantly, splenocytes from pSS mice are hyper-responsive to TLR2 ligation and female NOD.B10 salivary tissue shows heightened inflammatory cytokine secretion in the presence of LPS. In addition, production of IL-6, TNFα, and MCP-1 requires Myd88 in salivary tissue. Finally, Dcn may represent a novel mediator of TLR-induced inflammation in pSS, as this ECM molecule modulated production of inflammatory mediators in splenocytes. Thus, our work suggests an important role for activation of TLR2 and TLR4 both locally and systemically in pSS.

## Data Availability Statement

The datasets generated for this study can be found in the Gene Expression Omnibus (GEO) accession number GSE136402.

## Ethics Statement

The animal study was reviewed and approved by the University at Buffalo Institutional Animal Care and Use Committee.

## Author Contributions

JMK conceived of the work, wrote the manuscript, and performed experiments. JK and EK critically edited the manuscript and performed experiments. R-AR and GY critically edited the manuscript and analyzed the RNA-sequencing data.

### Conflict of Interest

The authors declare that the research was conducted in the absence of any commercial or financial relationships that could be construed as a potential conflict of interest.

## Supplementary Material

Supplemental Figure 1 Splenocytes were isolated from NOD.B10 females (*n* = 6) with clinical disease and age and sex-matched BL/10 controls (*n* = 3). Splenocytes were isolated from NOD.B10 females (*n* = 5) with clinical disease and age and sex-matched BL/10 controls (*n* = 5). Cells were cultured in media alone, Dcn and PMB, or *S. typhimurium* LPS for 24 h. Supernatants were harvested and **(A)** IL-1α, **(B)** IL-1β, **(C)** IL-2, **(D)** IL-4, **(E)** IL-5, **(F)** IL-10, **(G)** IL-12p70, and **(H)** IL-17 levels were quantified by cytokine multiplex array. All samples were evaluated in triplicate. Significance was determined using the Mann–Whitney test. Mean and SEM are shown (^**^*p* ≤ 0.01).
