# Supplementary figures and images for: Activation of Myd88-Dependent TLRs Mediates Local and Systemic Inflammation in a Mouse Model of Primary Sjögren's Syndrome
Source: Front Immunol. 2020 Jan 9;10:2963. doi: 10.3389/fimmu.2019.02963 (PMC6964703; doi:10.3389/fimmu.2019.02963)

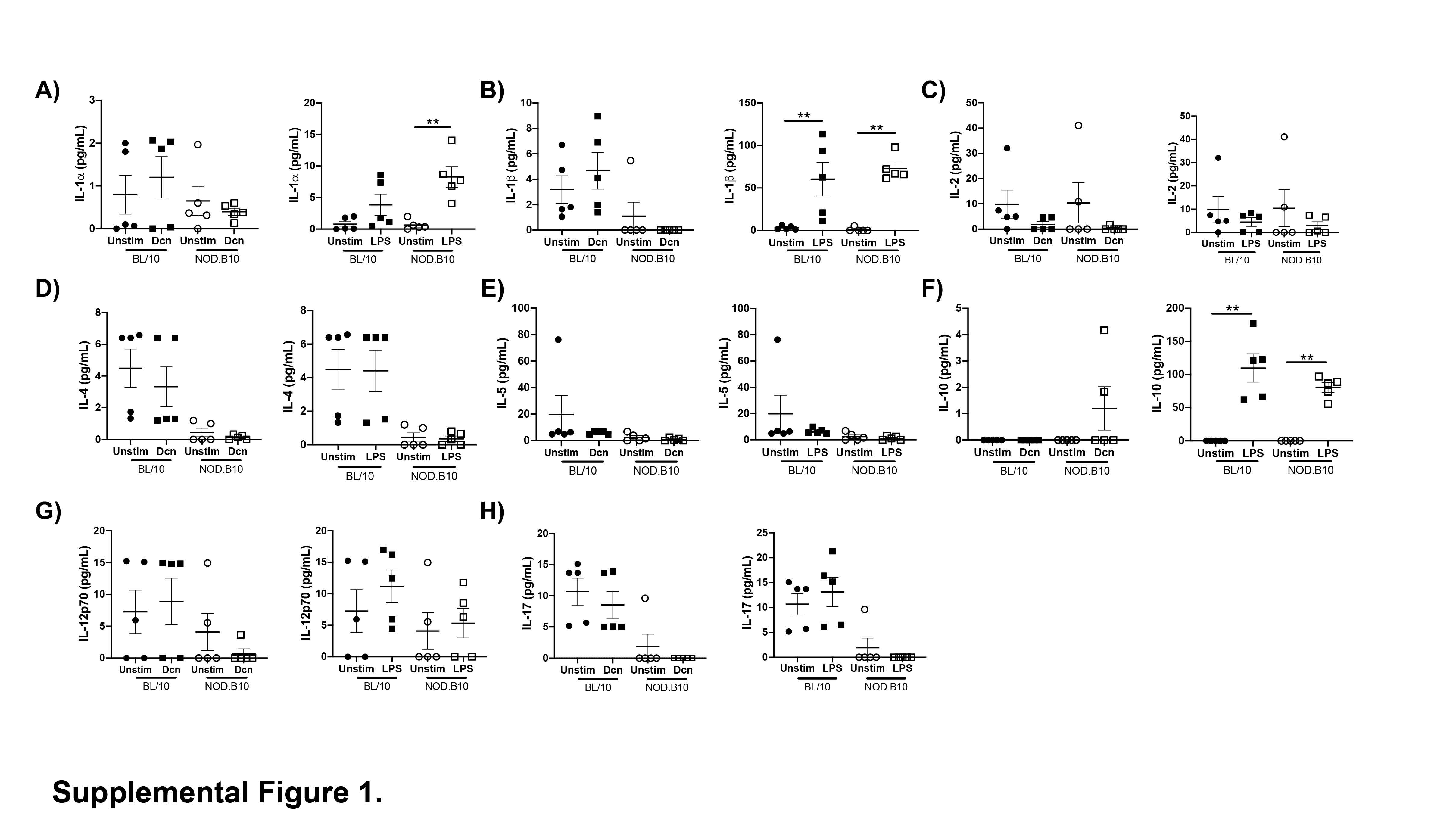

Supplement: Supplemental Figure 1 — Splenocytes were isolated from NOD.B10 females (n = 6) with clinical disease and age and sex-matched BL/10 controls (n = 3). Splenocytes were isolated from NOD.B10 females (n = 5) with clinical disease and age and sex-matched BL/10 controls (n = 5). Cells were cultured in media alone, Dcn and PMB, or S. typhimurium LPS for 24 h. Supernatants were harvested and (A) IL-1α, (B) IL-1β, (C) IL-2, (D) IL-4, (E) IL-5, (F) IL-10, (G) IL-12p70, and (H) IL-17 levels were quantified by cytokine multiplex array. All samples were evaluated in triplicate. Significance was determined using the Mann–Whitney test. Mean and SEM are shown (**p ≤ 0.01). [file Image_1.jpeg]
